# Case Report: A case of Dubin-Johnson syndrome in a newborn

**DOI:** 10.3389/fped.2024.1417649

**Published:** 2024-07-19

**Authors:** Junshan Long, Baowei Qiu, Xiaoxia Su, Jing Zhang, Qi Dong

**Affiliations:** Department of General Surgery, Hainan Women and Children’s Medical Center, Hainan Women and Children’s Medical Center, Haikou, Hainan, China

**Keywords:** Dubin-Johnson syndrome, *ABCC2*, mutation, newbown, jaundice

## Abstract

**Background:**

Dubin-Johnson Syndrome (DJS) is a rare autosomal recessive genetic disorder, with most cases presenting in adolescence, but rare in newborns.

**Objective:**

To investigate the clinical characteristics and treatment outcomes of DJS in a newborn.

**Methods:**

We present the clinical features of a newborn diagnosed with DJS through molecular genetic testing.

**Results:**

The patient was a male newborn who developed jaundice and scleral icterus on the 6th day of life. Both direct and indirect bilirubin levels were elevated. After treatment with phototherapy, indirect bilirubin levels decreased, but direct bilirubin remained unchanged, and the stool color gradually lightened. At 56 days of age, the patient underwent laparoscopic cholecystostomy, which revealed viscous bile plugs in the bile ducts. Following the surgery, the patient received oral ursodeoxycholic acid, compound glycyrrhizin, and methylprednisolone. Follow-up until one year post-surgery showed a gradual reduction in direct bilirubin levels to the normal range. Molecular genetic testing revealed three heterozygous mutations in the *ABCC2* gene on chromosome 10, with one pathogenic variant inherited from the father and two from the mother, confirming the diagnosis of DJS.

**Conclusion:**

DJS is a benign condition with a favorable prognosis. In newborns, it should be differentiated from other causes of cholestasis, and compared to cholestasis, jaundice in newborns with DJS responds more slowly to treatment.

## Introduction

Dubin-Johnson Syndrome (DJS) was first reported by Dubin and Johnson in 1954 (1). It is a rare autosomal recessive genetic disorder characterized by prolonged or intermittent jaundice (2). The prevalence of DJS varies among ethnic groups, with a higher incidence in the Han population. Numerous studies have documented this condition (3–6). DJS results from mutations in the ATP-binding cassette subfamily C member 2 (*ABCC2*) gene located on chromosome 10, leading to dysfunction of the multidrug resistance protein 2 (MRP2) (7).

DJS affects both males and females equally, with most cases presenting during adolescence, while it is rare in newborns (8). When DJS occurs in the neonatal period, other cholestatic problems must be ruled out first. Therefore, molecular genetic testing should be conducted in neonates to confirm the underlying cause. This article aims to discuss the clinical features and treatment outcomes of DJS by reviewing a case of DJS in a newborn.

## Clinical data and treatment course

**Figure 1:**
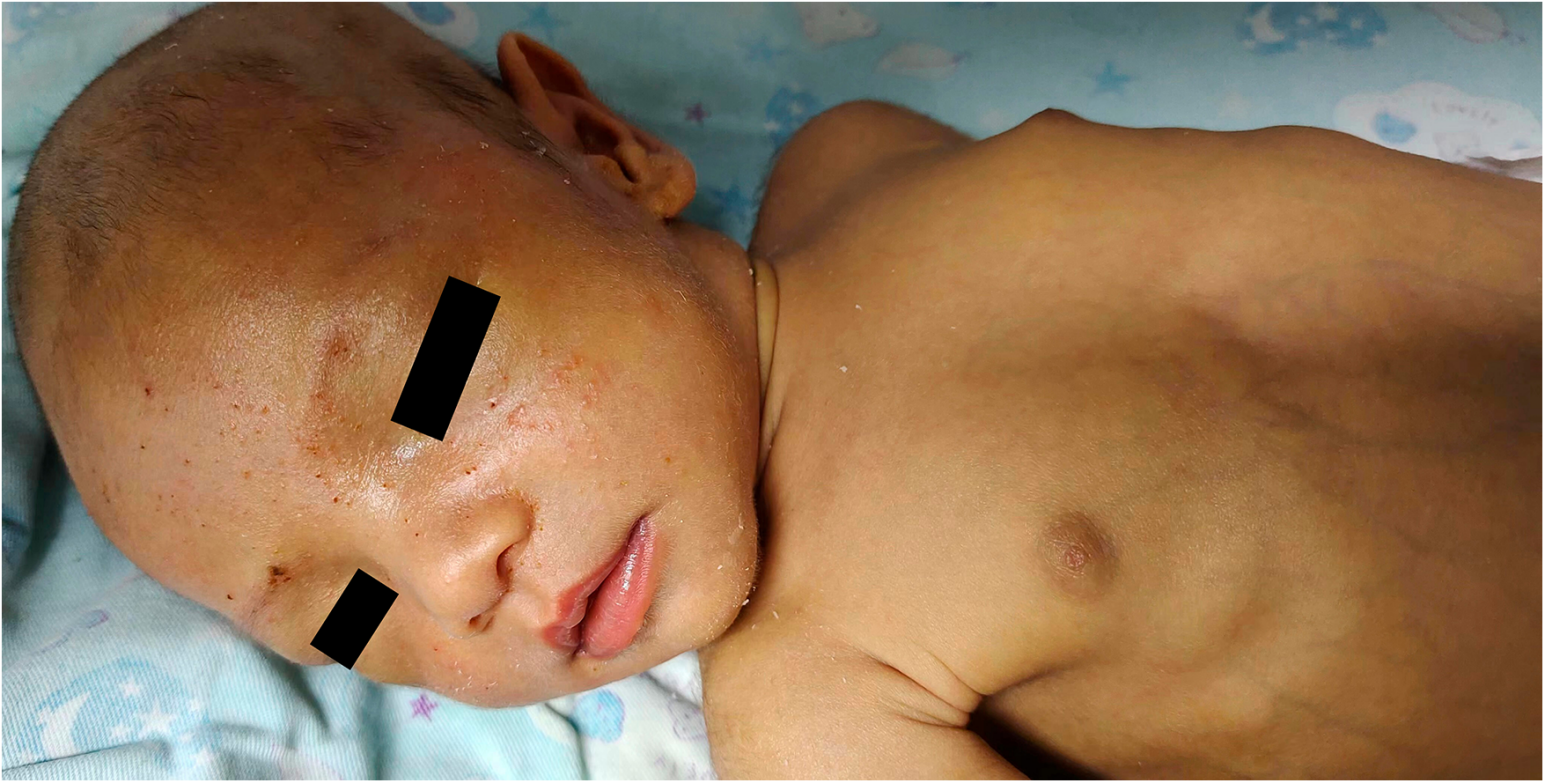
Patient's appearance at 53 days after birth. At 53 days after birth, the patient continued to exhibit persistent jaundice. Laboratory results showed total bilirubin 334 *μ*mol/L, direct bilirubin 260 *μ*mol/L, *γ*-glutamine transpeptidase 517 U/L, alkaline phosphatase 753 U/L, and total bile acids 184 *μ*mol/L.

The patient is a male, born as the first child to a healthy, non-consanguineous couple. There is no family history of similar illnesses or genetic disorders, and the patient's parents have no history of cholestasis. The patient was born at a gestational age of 39 + 6 weeks with a birth length of 47 cm and a weight of 2,750 g. On the 6th day after birth, the patient developed jaundice and scleral icterus, which gradually worsened, although the stool color remained normal. At 16 days of age, laboratory tests revealed the following results: total bilirubin 451 *μ*mol/L, direct bilirubin 168 *μ*mol/L, *γ*-glutamine transpeptidase 204 U/L, alkaline phosphatase 248 U/L, and total bile acids 112 *μ*mol/L. An abdominal ultrasound examination showed normal gallbladder size and contraction function, while a cardiac ultrasound revealed a 3.2 mm atrial septal defect. MRCP suggested the presence of extrahepatic bile ducts. The patient also presented with an umbilical hernia, bilateral hydroceles, and a left-sided cryptorchidism. Oral ursodeoxycholic acid and supplementation of fat-soluble vitamins did not reduce the jaundice, and the stool color gradually lightened to the shade of a No. 4 stool color card (9). On the 53rd day after birth (see Figure 1), The skin jaundice and sclera icterus did not improve, with total bilirubin decreasing to 334 *μ*mol/L, but direct bilirubin increasing to 260 *μ*mol/L. Additionally, γ-glutamine transpeptidase reached 517 U/L, alkaline phosphatase 753 U/L, and total bile acids 184 *μ*mol/L. On the 55th day after birth, the patient underwent laparoscopic cholecystostomy. During the surgery, the liver appeared black, with a normal gallbladder size and morphology. Washing the gallbladder revealed deep yellow, viscous bile plugs. Intraoperative imaging showed patent left and right hepatic ducts. Post-surgery, dexamethasone and saline were used to flush the cholecystostomy tube, resulting in a gradual deepening of stool color. Two weeks after the surgery, bilirubin levels began to decrease. One month post-surgery, the cholecystostomy tube was removed, and the skin jaundice and scleral icterus gradually faded. The patient received oral methylprednisolone for 6 months, and ursodeoxycholic acid and compound glycyrrhizin for 10 months.

**Figure 2:**
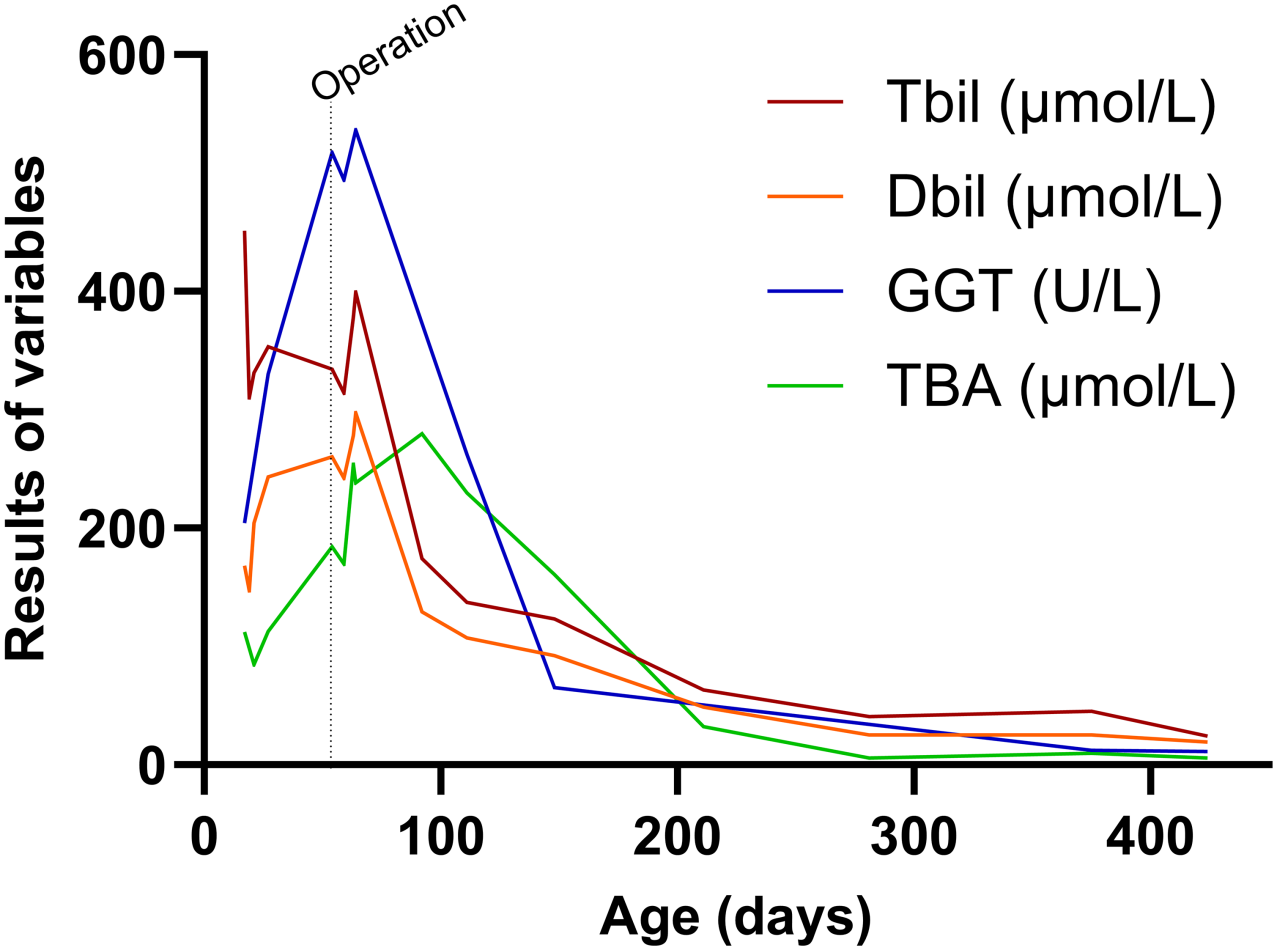
Changes in patient's liver function. At 55 days after birth, the patient underwent laparoscopic cholecystostomy. It was only two weeks after the surgery that the total bilirubin, direct bilirubin, and γ-glutamine transpeptidase levels began to decrease. One month after surgery, bile acid levels started to decrease, and after one year of follow-up, bilirubin levels returned to normal.

Follow-up until one year post-surgery showed that bilirubin levels had returned to normal (see Figure 2). Subsequently, the patient underwent laparoscopic surgery for left testicular descent fixation. During this procedure, the liver remained black with a normal size, firm texture, and smooth surface. The gallbladder had developed well, and the right internal ring was closed.

## Molecular genetic testing

In the case, a patient with unexplained cholestasis and significantly elevated direct bilirubin levels following birth did not respond well to drug treatment over a month. To explore the underlying causes and obtain a definitive diagnosis, exome sequencing was performed on the patient and both parents, with informed consent from the patient's parents.

**Figure 3:**
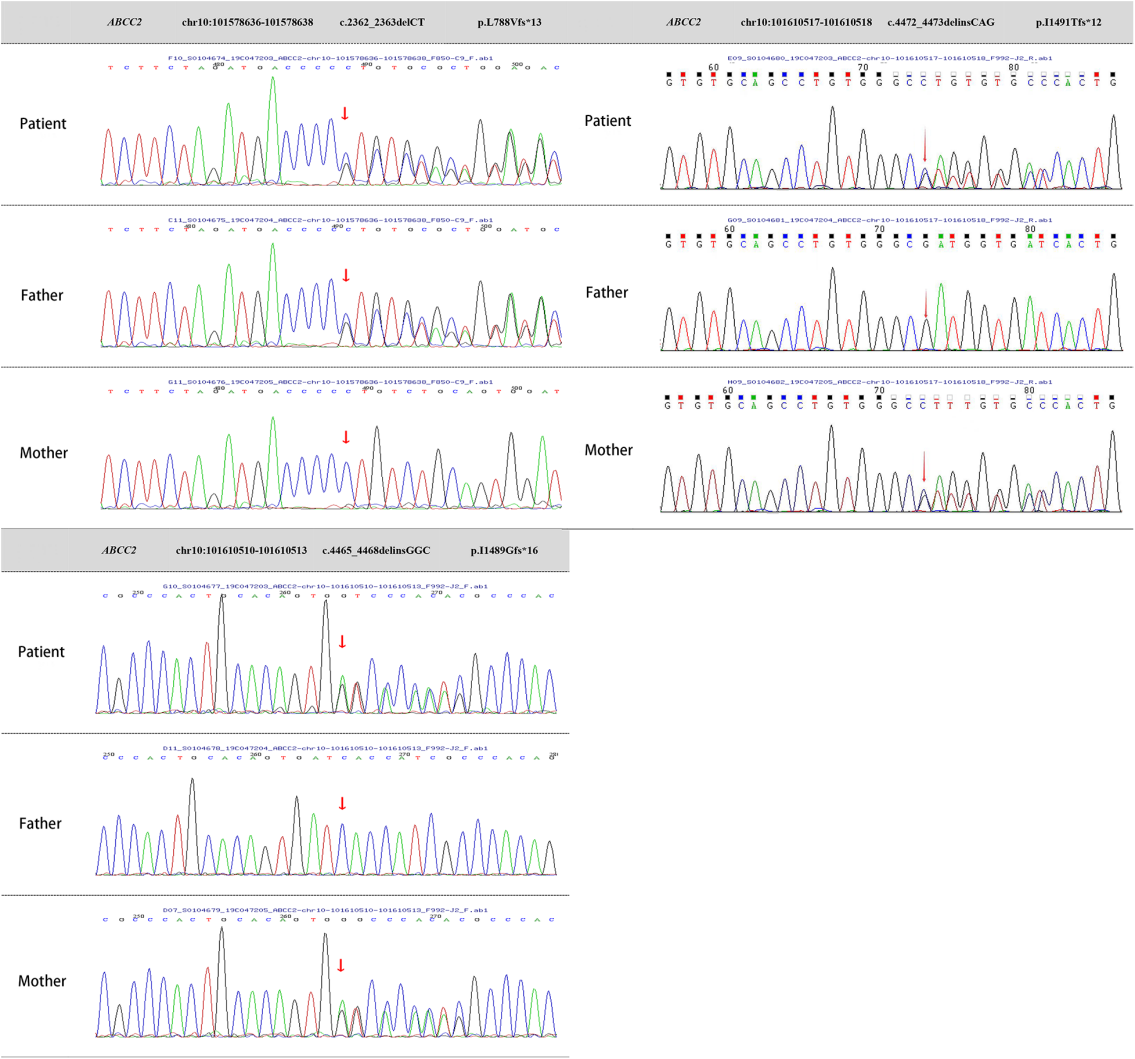
Genetic testing images of the patient and parents. *ABCC2* gene mutations:Heterozygous mutation c.2362_2363delCT, resulting in amino acid frameshift mutation (p.L788Vfs*13). Heterozygous mutation c.4465_4468delinsGGC, causing amino acid frameshift mutation (p.I1489Gfs*16). Heterozygous mutation c.4472_4473delinsCAG, leading to amino acid frameshift mutation (p.I1491Tfs*12).

Two milliliters of venous blood were collected separately from the patient and parents into ethylenediaminetetraacetic acid (EDTA) anticoagulant tubes. DNA was extracted using the QIAamp Whole Blood DNA extraction kit (Qiagen, Germany), yielding 3 *μ*g of DNA. Ultrasonic fragmentation was performed using the Covaris S2 ultrasonic instrument. Whole-genome libraries were prepared with the NEBNext DNA Library Prep Kit for Illumina (NEB, USA). GenCap liquid-phase target gene capture technology (Beijing MyGenostics Inc.) was applied (10). Testing revealed heterozygous mutations in the *ABCC2* gene on chromosome 10 (chr10: 101578636–101578638, chr10: 101610510–101610513, chr10: 101610517–101610518), resulting in nucleotide heterozygous mutations and corresponding amino acid frameshift mutations: c.2362_2363delCT (p.L788Vfs*13), c.4465_4468delinsGGC (p.I1489Gfs*16), and c.4472_4473delinsCAG (p.I1491Tfs*12). These mutation sites are indicated by arrows in Figure 3.

Sanger sequencing validation and pedigree analysis confirmed that the c.2362_2363delCT (p.L788Vfs13) heterozygous mutation originated from the father, while the c.4465_4468delinsGGC (p.I1489Gfs16) and c.4472_4473delinsCAG (p.I1491Tfs12) heterozygous mutations came from the mother. A search of the Human Gene Mutation Database and PubMed databases revealed that the c.2362_2363delCT (p.L788Vfs13) heterozygous mutation has been reported in malignancies but not in DJS. In contrast, the c.4465_4468delinsGGC (p.I1489Gfs16) and c.4472_4473delinsCAG (p.I1491Tfs12) heterozygous mutations had not been reported internationally, making them novel mutations. The mechanism by which *ABCC2* gene heterozygous mutations lead to DJS has been well-established, and based on the guidelines from the American College of Medical Genetics and Genomics, these mutations can be classified as pathogenic. Therefore, the patient's molecular genetic diagnosis was confirmed, resulting in a final diagnosis of DJS.

## Discussion

DJS is an autosomal recessive genetic disorder caused by the dysfunction of the MRP2 protein. It can affect individuals of all ethnicities and genders, with males often manifesting the condition earlier (11, 12). DJS typically does not progress to fibrosis or cirrhosis and does not require any specific treatment. However, in cases of severe cholestasis in newborns with DJS, prolonged intrahepatic pigment deposition can lead to hepatocyte degeneration, necrosis, fibrosis, and other pathological changes. In such instances, it is crucial to actively manage jaundice, protect the liver, and reduce enzyme levels to minimize clinical symptoms and liver cell damage (13). Treatment may involve the use of phenobarbital and ursodeoxycholic acid in newborns (14).

In this case report, the patient developed skin jaundice just 6 days after birth, with elevated serum bilirubin levels, consistent with the typical presentation of DJS. Due to the dysfunction of MRP2 protein, which leads to hepatic impairment, the patient's stool color gradually lightened, and the levels of *γ*-glutamine transpeptidase increased, indicating the blocked bile excretion (15). Due to the persistent jaundice, surgical treatment was ultimately performed. The patient underwent laparoscopic cholecystostomy and received ursodeoxycholic acid and steroid treatment post-surgery. Bilirubin levels began to decrease two weeks after surgical intervention, indicating a slower response to jaundice resolution in newborns with DJS compared to others cholestasis.

Genetic testing has become essential for diagnosing DJS and distinguishing newborn cholestasis conditions (16). In this case, the patient had heterozygous mutations in the *ABCC2* gene on chromosome 10, with one pathogenic variant inherited from the father and two from the mother. Interestingly, despite carrying two ABCC2 gene mutations, the mother has no history of cholestasis. Additionally, novel mutations c.4465_4468delinsGGC (p.I1489Gfs*16) and c.4472_4473delinsCAG (p.I1491Tfs*12) were identified. The absence of prior reports on these mutations suggests the genetic heterogeneity of DJS, which may contribute to differences in disease progression and treatment response among patients. Both mutations are located on the same chromosome, with the first mutation (p.I1489Gfs*16) primarily causing the loss of protein function, which also affects the function of the second mutation (p.I1491Tfs*12). The ABCC2 gene is primarily expressed in the hepatocyte canalicular membrane. The patient's three different mutations in the ABCC2 gene affect the excretion of bilirubin into the bile and limit the sulfation of bile salts. Consequently, normal liver function is impaired, leading to reduced synthesis of bilirubin conjugates (glucuronides) and elevated bilirubin levels (17, 18). However, the mild hepatic phenotype and lack of extrahepatic symptoms in ABCC2 deficiency suggest that other transporters can compensate for its function (17).

Some studies have reported that phenobarbital and ursodeoxycholic acid treatment in DJS newborns can lead to a gradual reduction in bilirubin levels (16). Another study highlighted that hepatoprotective and choleretic treatment in three DJS newborns resulted in improved liver function, reduced transaminase, and bilirubin levels, and a favorable prognosis (13). However, In this case, conservative treatment prior to surgery proved to be less effective, prompting surgical intervention in the absence of excluded biliary atresia. Post-surgery, jaundice gradually receded, contrasting with the findings of the earlier studies. Although DJS is considered a benign condition that typically requires no special treatment, we administered medication to the patient in this case to alleviate the caregivers' anxiety (17).

DJS is generally considered a benign condition with mild clinical symptoms and a favorable prognosis. However, in cases of severe jaundice in newborns with DJS, active treatment is essential to protect the liver.

## Data Availability

The raw sequence data reported in this paper have been deposited in the Genome Sequence Archive (Genomics, Proteomics & Bioinformatics 2021) in National Genomics Data Center (Nucleic Acids Res 2022), China National Center for Bioinformation / Beijing Institute of Genomics, Chinese Academy of Sciences (GSA-Human: HRA006234) that are publicly accessible at https://ngdc.cncb.ac.cn/gsa-human.
